# Structure of a Cyclic Peptide as an Inhibitor of *Mycobacterium tuberculosis* Transcription: NMR and Molecular Dynamics Simulations

**DOI:** 10.3390/ph17111545

**Published:** 2024-11-18

**Authors:** Filia Stephanie, Usman Sumo Friend Tambunan, Krzysztof Kuczera, Teruna J. Siahaan

**Affiliations:** 1 Department of Pharmaceutical Chemistry, School of Pharmacy, The University of Kansas, Lawrence, KS 66047, USA; 2 Department of Chemistry, University of Indonesia, Depok 16424, Indonesia; usman@ui.ac.id; 3 Department of Chemistry, The University of Kansas, Lawrence, KS 66045, USA; kkuczera@ku.edu; 4 Department of Molecular Biosciences, The University of Kansas, Lawrence, KS 66045, USA

**Keywords:** cyclic peptide conformation, molecular dynamics simulation, mRNA polymerase inhibitor, *Mycobacterium tuberculosis*, nuclear magnetic resonance

## Abstract

Background and Objectives: A novel antitubercular cyclic peptide, Cyclo(1,6)-Ac-CLYHFC-NH_2_, was designed to bind at the rifampicin (RIF) binding site on the RNA polymerase (RNAP) of *Mycobacterium tuberculosis* (MTB). This peptide inhibits RNA elongation in the MTB transcription initiation assay in the nanomolar range, which can halt the MTB transcription initiation complex, similar to RIF. Therefore, determining the solution conformation of this peptide is useful in improving the peptide’s binding affinity to the RNAP. Methods: Here, the solution structure of Cyclo(1,6)-Ac-CLYHFC-NH_2_ was determined by two-dimensional (2D) NMR experiments and NMR-restrained molecular dynamic (MD) simulations. Results: All protons of Cyclo(1,6)-Ac-CLYHFC-NH_2_ were assigned using TOCSY and NOE NMR spectroscopy. The NOE cross-peak intensities were used to calculate interproton distances within the peptide. The J_NH-HCα_ coupling constants were used to determine the possible Phi angles within the peptide. The interproton distances and calculated Phi angles from NMR were used in NMR-restrained MD simulations. The NOE spectra showed NH-to-NH cross-peaks at Leu2-to-Tyr3 and Tyr3-to-His4, indicating a βI-turn formation at the Cys1-Leu2-Tyr3-His4 sequence. Conclusions: The NMR-restrained MD simulations showed several low-energy conformations that were congruent with the NMR data. Finally, the conformation of this peptide will be used to design derivatives that can better inhibit RNAP activity.

## 1. Introduction

Tuberculosis (TB) is a leading cause of death worldwide and remains a global problem [1,2,3]. Although it is a treatable disease, the complex drug regimen and drug resistance issues are becoming a massive obstacle in the treatment of this infectious disease [4,5,6,7,8]. The repeated use of antibiotics for TB treatment created drug resistance problems; therefore, there is a need to develop alternative therapeutic agents for the disease [4,7,8,9,10,11]. Rifampicin (RIF) is a first-line drug for TB, alongside isoniazid (INH) [7,10,12,13,14]. RIF inhibits the RNA polymerase (RNAP) activity in *Mycobacterium tuberculosis* (MTB). Therefore, designing and developing therapeutic peptides and peptidomimetics that bind to the RIF binding site could increase the number of alternative drugs for TB to overcome drug resistance [11,13].

Using computational screening, we designed six cyclic peptides that can potentially bind to the RIF binding sites at the MTB RNAP [13]. These cyclic peptides were selected from 7500 peptide sequences generated using amino acid combinations that can bind to the RIF binding site. These six peptides were previously synthesized and evaluated for their activity to inhibit MTB RNA polymerase. Cyclo(1,6)Ac-CLYHFC-NH_2_ was found to inhibit RNA elongation in the nanomolar range as determined by the MTB transcription initiation assay. Similar to RIF, inhibiting MTB RNA polymerase activity can halt MTB transcription initiation [15,16,17,18].

Peptide conformational studies are extensively used in drug discovery to identify lead compounds, active peptide fragments, structure–activity relationships, and bioactive conformations [19,20,21]. Peptide conformation has been correlated with the transport properties of peptides across cell membranes for cell uptake as well as peptide absorption through biological barriers such as the intestinal mucosa and the blood–brain barrier (BBB) [17,18,22,23]. It has been shown previously that cyclic peptides have better membrane transport properties than their parent linear peptides [17]. This is due to the stabilized conformation of the cyclic peptide compared to the parent linear peptide. Additionally, cyclization of linear peptide alters its physicochemical properties such as hydrogen bonding potential with water, intramolecular hydrogen bonds, secondary structure, and hydrophobicity (LogP) [17,22,23]. Therefore, studying the peptide conformation can help design peptide derivatives that have high enzyme-binding properties with the ability to penetrate cell membranes.

In this study, we determined the solution conformation of Cyclo(1,6)Ac-CLYHFC-NH_2_ using NMR and molecular dynamics (MD) simulations. Each proton within the peptide was identified using COSY, TOCSY, and NOESY NMR experiments [20,24,25,26]. Linear peptides can adopt an ensemble of conformations in solution, resulting in bioactivity against diverse targets. Unlike flexible linear peptides, cyclic peptides have a more restricted conformation, thus resulting in higher structural rigidity and greater specificity towards the target receptor [27,28]. Therefore, the interproton distances within this cyclic peptide were determined from the cross-peak volumes of proton–proton interactions in the NOESY spectra. The NH-to-HCα coupling constants (J_NH-HCα_) were used to determine the Phi dihedral angles within the peptide [29]. The NH temperature dependence was evaluated to determine NH protons that are involved in intramolecular hydrogen bonding [30]. The interproton distances and Phi dihedral angles were used as restraints in MD simulations to find the preferable conformation Cyclo(1,6)Ac-CLYHFC-NH_2_ peptide in solution.

## 2. Results

### 2.1. Chemical Shift Assignment

**Figure 1:**
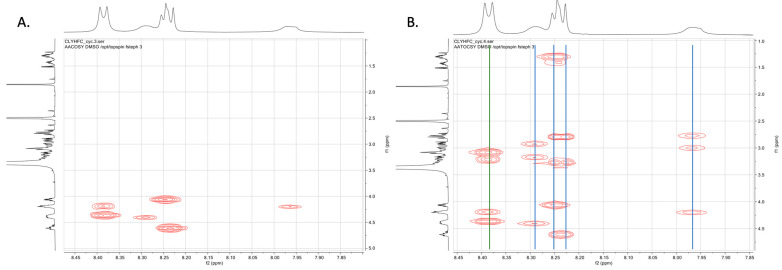
(**A**) COSY and (**B**) TOCSY spectra of Cyclo(1,6)-Ac-CLYHFC-NH_2_ in DMSO-d_6_ at 298 K using a 500 MHz Bruker AVIII spectrometer (Billerica, MA, USA). From the TOCSY spectra, 5 NH spin systems were identified, as denoted with vertical lines. Blue vertical lines denote spin system of a single amino acid residue, while the green vertical line denotes two spin systems belonging to two different residues in complete overlap with each other.

The 1D and 2D NMR spectra of the Cyclo(1,6)Ac-CLYHFC-NH_2_ peptide showed high purity of the peptide observed from the absence of impurities peak identified. There was no observed precipitate or change in visual characteristics throughout the duration of the NMR experiments, indicating the stability of this peptide inside the solution. A conventional assignment strategy was carried out with 2D NMR to determine each proton in the Cyclo(1,6)-Ac-CLYHFC-NH_2_ peptide. First, we identified the spin system through the COSY and TOCSY spectra (Figure 1). The COSY spectrum showed six cross-peaks, and each cross-peak belongs to a single correlation between the amide (NH) and carbon alpha proton (HCα) of each residue (Figure 1A). From this spectrum, it could be deduced that a doublet peak downfield at 8.39 ppm corresponds to two amide protons from two residues overlapping with each other. The peaks at 8.32–8.21 ppm with multiplet were derived from three amide protons. From the TOCSY spectra (Figure 1B), five visible spin systems were identified, in which the first spin system downfield belongs to two residues. The Leu2 spin system was easily distinguished via the cross-peaks at the aliphatic region (1.1–1.3 ppm); this confirms the presence of Leu2 side chain protons. Furthermore, a complete assignment to the proton peaks was performed using a combination of COSY, TOCSY, and NOESY spectra.

To aid the assignment, ^1^H–^13^C HSQC and HMBC spectra were acquired to identify the resonance of the beta, gamma side-chain proton, as well as the acetyl group on the *N*-terminus. To confirm the cyclization, we obtained NMR spectra of the linear Ac-CLYHFC-NH_2_ peptide to identify the presence of sulfhydryl protons. In the ^1^H–^13^C HMBC spectrum, a cross-peak belonging to the correlation between the beta proton of Cys6 and the Cys6 sulfhydryl proton was identified, which was absent in the ^1^H–^13^C HMBC spectrum of the cyclic peptide (Figure S7). The beta protons from both Cys1 and Cys6 of the cyclic peptide were also found to be shifted downfield compared to the Cys HCβ protons on the linear peptide, which was also observed in NMR characteristics of the oxidized/reduced state of Cys in a random coil [31]. A broad peak at around ~14 ppm was observed for all NMR proton spectra for both linear and cyclic peptides. The ^19^F NMR experiment confirmed that this broad peak belongs to the residual trifluoro acetic acid (TFA) in the sample. This is because all synthetic peptides were obtained from purification using solvents containing TFA as a buffer Overall, amide protons were found in the range of 8.38–7.96 ppm, and HCα protons were at 4.62–4.06 ppm. The complete proton assignment of Cyclo(1,6)Ac-CLYHFC-NH_2_ at 298 K is tabulated in Table 1.

### 2.2. NOE Space Connectivity

**Figure 2:**
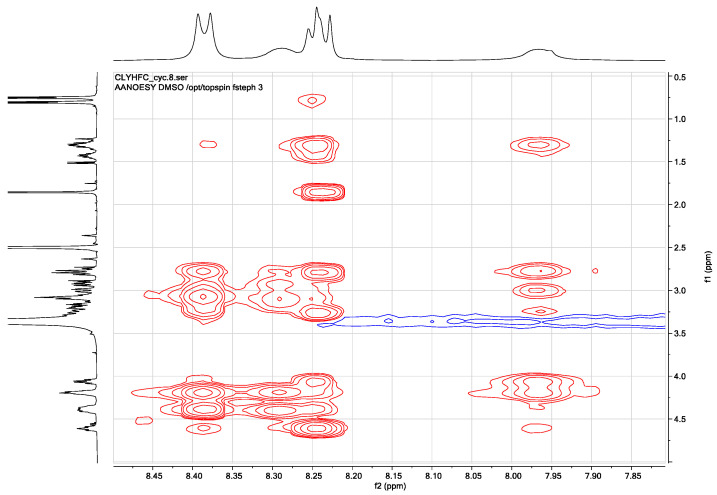
NOESY fingerprint region of Cyclo(1,6)Ac-CLYHFC-NH_2_ in DMSO-d_6_. The spectrum was acquired at 293 K using a 500 MHz Bruker AVIII spectrometer.

**Figure 3:**
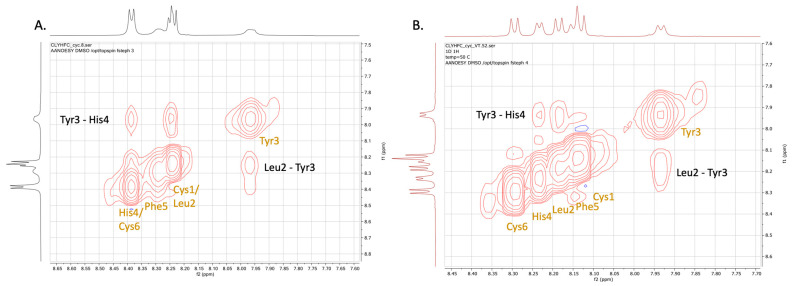
The NH-NH sequential connectivity in NOESY spectra show the Leu2-Tyr3 and Tyr3-His4 interactions observed at different temperatures: (**A**) 293 K and (**B**) 323 K.

The NOESY spectrum was used to confirm the proton assignments by COSY and TOCSY spectra (Figure 2). In addition, NOE cross-peaks were used to determine through space proton–proton interactions between (i, i), (i, i + 1), and (i, i + 2) residues. Firstly, sequential connectivity and secondary structure information can be determined from the NH-NH region of the NOESY spectrum. In the NH-NH region, the NH protons of His4 and Cys6 were overlapped as well as the NH protons of Cys1 and Leu2 (Figure 3A). To distinguish the overlapping NH protons, NOESY spectra were acquired at a higher temperature (323 K) (Figure 3B). At this temperature, the NH of His4 was separated from the NH of Cys6, while the NH of Cys1 was separated from the NH of Leu2. There were interactions between the NH of Leu2 and the NH of Tyr3 as well as between the NH of Tyr3 and the NH of His4. These sequential interactions indicate the presence of a βI-turn at the Cys1-Leu2-Tyr3-His4 sequence.

**Figure 4:**
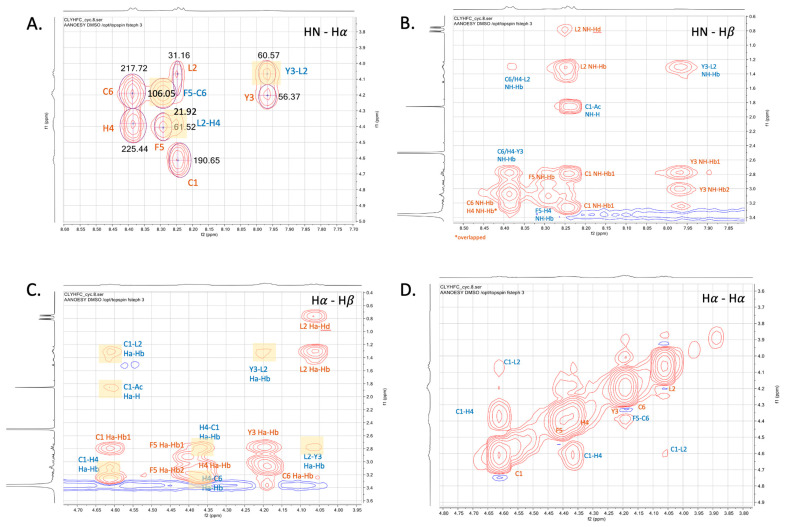
Through space connectivities in Cyclo(1,6)-Ac-CLYHFC-NH_2_ shown by NOE cross-peaks at (**A**) the NH-HCα region, (**B**) the NH-HCβ region, (**C**) the HCα-HCβ region, and (**D**) the HCα-HCα region. All spectra were acquired in DMSO-d_6_ at 298 K.

Many other inter-residue correlations were identified through various parts of the NOESY spectra. The NH (i) and HCα (i + 1) short range through space interactions were found in Phe5-Cys6 and Leu2-Tyr3. Similarly, a medium-range NOE (i, i + 2) between the NH of Leu3 (i) and the HCα of His4 (i + 2) was also observed (Figure 4A). The region showed interactions between the NH of Leu2 and HCβ of Tyr3; the NH of His4 and HCβ of Phe5; the NH of Leu2 and HCβs of His4/Cys6; and the NH of Tyr3 and HCβs of His4/Cys6 (Figure 4B). Medium-range interactions were found in cross-peaks between the HCα of Cys1 and the HCβ of Leu2; the HCα of Leu2 and the HCβ of Tyr3; and the HCα of Cys1 and the HCβ of His4 (Figure 4C). Another medium-range connectivity was found in cross-peaks between the HCα of Cys1 and the HCα of His4; the HCα of Cys1 and the HCα of Leu2; and the HCα of Phe5 and the HCα of Cys6 (Figure 4D).

### 2.3. Temperature-Dependent NMR

**Figure 5:**
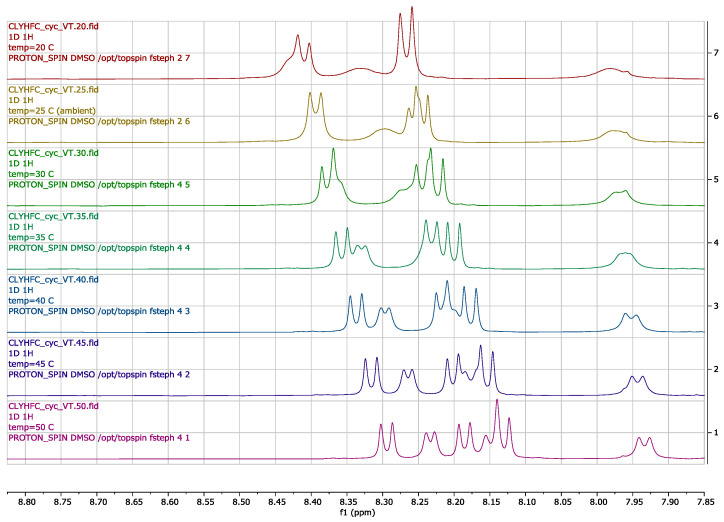
Temperature-dependent amide proton shift of Cyclo(1,6)Ac-CLYHFC-NH_2_. The experiment was conducted at the 293 K (red spectrum) to 323 K (purple spectrum) temperature range with 5 K increments.

To investigate the possibility of intramolecular hydrogen-bonding formation within the Cyclo(1,6)-Ac-CLYHFC-NH_2_ structure, a variable-temperature NMR experiment was performed on the peptide solution in DMSO-d_6_ (Figure 5). The experiment was performed at a temperature range of 293–323 K with a 5 K increment, using a single peptide sample. The lowest temperature was chosen according to the relatively high DMSO-d_6_ freezing point (291.5 K). As the temperature increased, the amide protons were shifted upfield; towards the end of the experiments, almost all amide proton peaks were resolved as doublet peaks. The resolved NH peaks aided the complete assignment of all protons within the peptide. Temperature coefficients for each amide proton were obtained by fitting the chemical shift to a linear model.

The effect of temperature on the NH chemical shift was evaluated to determine its temperature coefficient in ppb/K, which is correlated to the intramolecular hydrogen bond. The slope from a linear fitting of the chemical shift data vs. temperature for the NH of each residue is shown in Table 2. The NH of Tyr3 has a low temperature coefficient (1.64 ppb/K), indicating the possibility of the formation of a strong intramolecular hydrogen bond. The second lowest temperature coefficient was from the NH of Leu2 at 2.56 ppb/K. The low temperature coefficient correlates with the non-solvent accessibility of the amide proton due to a local contribution in shielding within the peptide structure.

### 2.4. Interproton Distance and Dihedral Angle Restraints

Interproton distances can be derived from NOE data to establish conformational details of Cyclo(1,6)Ac-CLYHFC-NH_2_. A total of 43 NOEs were assigned from NOESY spectra in DMSO-d_6_ at 293 K. These data consist of 14 sequential NOE including Cys-1 connectivity with the Ac group at the *N*-terminus with five medium range connectivity (2 (i, i + 2), 3 (i + 3) data). NOE cross-peak intensities were converted to interproton distances using Equation (1) with a reference distance of 1.78 Å for the intensity of the HCβ geminal protons of the Phe5 residue. The calculated interproton distances as NMR constraints are listed in Table 3.

To obtain the dihedral angle restraints, we determined the vicinal ^3^J_NH-HCα_ coupling constant from the NMR spectrum using the Mestrenova 14.2.0 software. The measured coupling constants were between 5.12 and 8.51 Hz for all residues, with Phe5 as the exception. The calculation for Phe5 was not possible due to the position of the amide proton peak that was buried within the multiplet without any defined peak/shoulder. The coupling constants were then used to derive all φ torsion angles possible for restraints on each peptide bond, as shown in Table 4.

### 2.5. Molecular Model of Cyclo(1,6)Ac-CLYHFC-NH_2_

To model the cyclic peptide, MD simulations were conducted with and without the NMR restraints. For the NMR-restrained MD simulation, NOE-derived interproton distances and Phi dihedral angles were introduced to the system. Positional restraints were applied to the backbone amide and alpha protons for computing efficiency. The calculated interproton distance was integrated as a range with upper bound and lower bound of ±1 Å from the experimental value.

**Figure 6:**
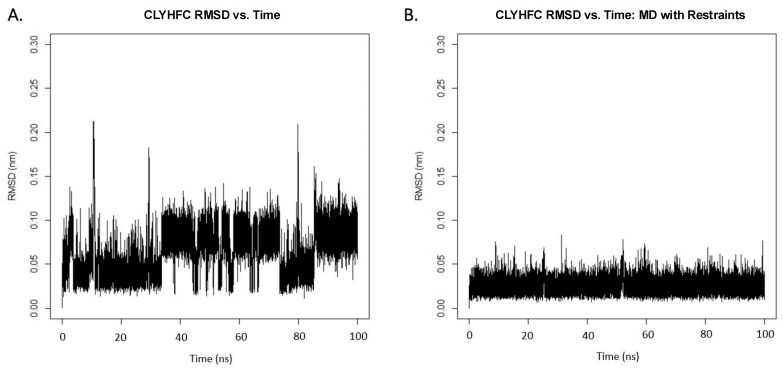
RMS deviation of the atomic position of Cyclo(1,6)Ac-CLYHFC-NH_2_ during 100 ns of MD simulation at 298 K with (**A**) no positional restraints and (**B**) NMR-derived restraints introduced.

**Figure 7:**
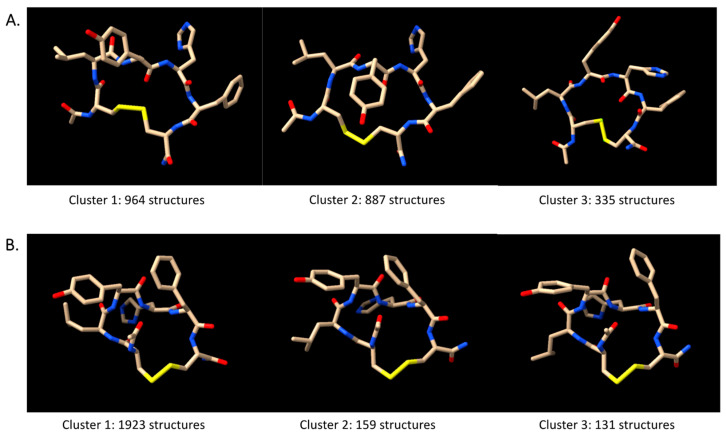
Top 3 clusters of (**A**) relaxed-state MD and (**B**) NMR-restrained MD simulation of Cyclo(1,6)Ac-CLYHFC-NH_2_ at 298 K, obtained using the *gmx cluster* module. The oxygen, nitrogen, and sulfur atoms are colored red, blue, and yellow, respectively.

For dihedral angles, we included the calculated φ angles for Leu2, Tyr3, and His4 as restraints on the topology file. After the 100 ns trajectory was obtained, we conducted the RMSD calculation for the system (Figure 6). It was observed that the positional restraints parameter effectively reduced the conformational space of the peptide, and representative structures with a lower deviation of the atomic position were visualized. Furthermore, clustering analysis was performed to obtain the molecular model from the MD trajectories. We extracted 2500 PDB snapshots from the 100 ns trajectories and used the GROMACS clustering algorithm to categorize the structures based on their conformational similarity using a 0.05 Å cut-off. The top three clusters from each simulation (free MD and NMR-restrained MD) are visualized in Figure 7.

From the model, the top cluster of the NMR-restrained simulation covers 1923 structures, around 77% of the total structures used from clustering. This means that this conformer persists throughout the simulation and thus can be obtained as one of the representative solution structures. Clusters found from the relaxed MD simulation resulted in more varied conformers. Distance analysis confirmed that the NMR-restricted simulation produced conformers with interproton distances that fall within the applied threshold for all three clusters. For instance, the inter-residue HN protons from Leu3 and Tyr3 were measured at 2.119 Å, 2.108 Å, and 2.298 Å in the top conformers of clusters 1, 2, and 3, respectively. Additionally, the HN protons from Tyr3 and His4 were at distances of 2.000 Å, 1.721 Å, and 2.057 Å in the corresponding clusters.

**Figure 8:**
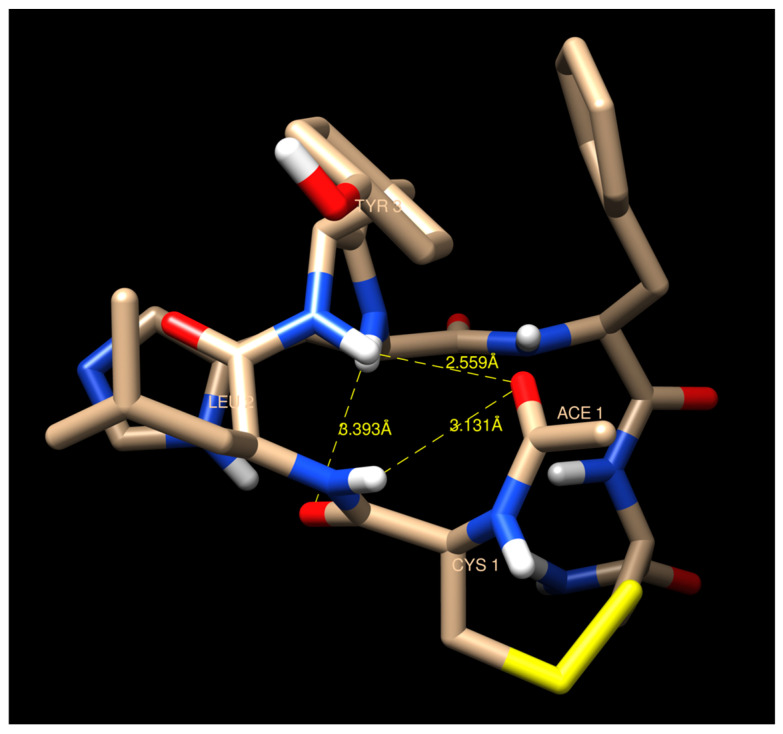
Distance calculation of Leu2 and Tyr3 amide protons with neighboring carbonyl oxygen in the top cluster from the NMR-restrained MD simulation of Cyclo(1,6)Ac-CLYHFC-NH_2_ at 298 K. The hydrogen, oxygen, nitrogen, and sulfur atoms are colored white, red, blue, and yellow, respectively.

Analysis of the intramolecular hydrogen bonding formation was performed with respect to the NMR-derived temperature coefficient. From the top clusters, we observed the distances between the amide protons and the neighboring hydrogen bond acceptor. Leu2 and Tyr3 amide protons were in proximity with the carbonyl oxygen of the *N*-terminus acetyl throughout the course of the simulation (3.131 Å and 2.559 Å), suggesting the formation of hydrogen bonds between the atoms with a 3.5 Å cut-off [32]. Additionally, the Tyr3 amide proton was also capable of forming an interaction with Cys1 carbonyl oxygen with a 3.393 Å distance on the top cluster. This supports the observation of the suppressed temperature coefficient for these two residues. Unlike the restrained model, the acetyl group on the unrestrained model was positioned outward from the main chain, resulting in a further distance from any of the hydrogen donor groups. The formation of hydrogen bonding on the top cluster is visualized in Figure 8.

The phi and psi angles from the top models obtained through the NMR-restrained and unrestrained simulation were measured and compared, as shown in Table 5. The phi angles of Cys1 and psi angles of Cys6 were excluded due to a geometry change from *N*-terminus acetylation, C-terminus amidation, and Cys side chain cyclization. From the result, it was observed that the restrained model has close values with the calculated angles from the coupling constants, unlike the unrestrained model with higher deviations. All peptide bonds were observed in the trans configuration, with a measured ω angle of ~180°.

## 3. Discussion

Currently, more than 70 peptides have been approved to treat various diseases, and many more peptides are in clinical trials for approval as therapeutic and diagnostic agents. Many of these peptide drugs are cyclic in nature. Peptides possess various advantages as therapeutics, including (a) high affinity to cell surface receptors, (b) low immunogenicity, and (c) higher synthetic accessibility compared to other classes of compounds. In addition, peptides are tailorable to occupy a larger contact area on target receptors compared to small molecule drugs. Despite their promising properties, peptides are known to generally have poor oral bioavailability due to their poor membrane permeability and susceptibility to enzyme degradation in vivo. Cyclic peptides have improved membrane permeation compared to their parent linear peptides; thus, they have better transport properties across biological barriers (i.e., intestinal mucosa barrier, blood–brain barrier (BBB)) [17,18,22,23]. This is due to the improvement in the physicochemical properties of cyclic peptides compared to linear peptides. Cyclic peptides form intramolecular hydrogen bonding to decrease hydrogen bonding potential to water compared to linear peptides [17,22,23]. In addition, the formation of cyclic peptides can increase hydrophobicity (i.e., LogP) and decrease molecular size to favor cell membrane permeation compared to the respective linear peptides. The plasma stability of cyclic peptides is higher than parent linear peptides because the rigid conformation of cyclic peptides reduced their recognition as a substrate for endo- and exo-peptidases in plasma.

In a previous study, we found that Cyclo(1,6)Ac-CLYHFC-NH_2_ inhibited RNA polymerase activity. In this study, we determined the conformation of this peptide that will be used to design derivatives with improved biological activity, bioavailability, and selectivity. In solution, peptide conformations always exist in an ensemble rather than a single rigid conformation. Different conformations of peptides have different efficiency in recognizing a specific target receptor. As an example, a certain conformation of RGD peptide can target a certain integrin receptor [30]. Thus, MD simulation was used to sample the conformation ensembles of a cyclic peptide. Due to its flexible nature and large conformational space, it is extremely complicated to identify a subset of biologically relevant conformers of a peptide. Thus, to enhance the modeling efficiency, interproton distance, and dihedral angle restraints, NMR experiments were incorporated into the simulation to determine the peptide solution conformation.

Here, interproton distances from NOE spectra were used in MD simulations to determine the peptide conformations. There are clear differences in the structural clusters of non-restrained dynamics and the NMR-restrained dynamics (Figure 6). One model from the top cluster has fewer violations towards the restraints compared to the other two models. The NMR-restrained MD simulations showed a βI-turn at the Cys1-Leu2-Tyr3-His4 sequence, as supported by the NH–NH interactions between Leu2 and Try3 as well as between Tyr3 and His4. Temperature-dependent NMR experiments for the amide proton chemical shift were conducted to analyze the occurrence of hydrogen bonding network within the molecule and identify exposed amide proton. The temperature coefficients of the NHs of Leu2 and Tyr3 were low, and the structural models showed that these NH protons were buried in the βI-turn structure. The NHs of Leu2 and Tyr3 can form H-bonding to the carbonyl of the *N*-terminal acetyl group and the Cys1 residue. The temperature-dependent NMR experiment did not show a low temperature coefficient of the NH of His4; thus, the NH of His4 as residue (1 + 3) did not form a hydrogen bond to the carbonyl of Cys-1 as residue (i) in a traditional definition of a βI-turn. The relaxed MD simulations showed that the NH of His4 did not form a H-bond to the CO of Cys1 with a distance of 6.6623 Å (Figure 7A). In contrast, the NMR-restrained MD simulations could form a H-bond between the NH of His4 and the CO of Cys1 with a distance of 2.6135 Å (Figure 7B) [33]. This result suggests that the His4 residue is in a more flexible C-terminal region while the *N*-terminus of the peptide is in a more rigid or locked βI-turn conformation. In addition, the strong H-bonds between the HN of Tyr3 and the CO of Cys1 and the NH of Leu2 and the CO of the acetyl group could prevent the formation of the H-bond between the NH of His4 and the CO of Cys1.

In the future, the solution conformation of Cyclo(1,6)Ac-CLYHFC-NH_2_ will be used to design derivatives that have better activity to inhibit MTB RNA. Structural studies have been shown to be beneficial to explore structure–activity relationships. Previously, cyclic peptides were used as effective drugs for patients such as oxytocin, integrilin, and octreotide [34]. Integrilin is a cyclic RGD peptide that selectively binds to the gpIIb/IIIa integrin receptor on the surface of platelets to inhibit platelet aggregation; therefore, integrilin is used as a drug for thrombosis in patients [35]. Furthermore, Aggrastat^TM^ is an RGD peptidomimetic; this peptidomimetic was designed based on the structure of cyclic RGD peptides that are selective for the gpIIb/IIIa integrin receptor [35]. Similarly, cyclic RGD peptides that are selective for α_v_β_3_ and α_v_β_5_ integrins have been used to inhibit angiogenesis; therefore, these cyclic RGD peptides have been used to suppress tumor growth [35]. Furthermore, as this study highlights the structural features of this cyclic peptide, future studies may include derivatization and structure–activity relationship studies with the receptor. Structural and dynamics characterization will also be performed against the mutant receptor to address the drug resistance issue of TB.

## 4. Materials and Methods

### 4.1. Peptide Synthesis

Cyclo(1,6)Ac-CLYHFC-NH_2_ was synthesized using a combination of solid and solution phase chemistries as described previously by Stephanie et al. [13]. Briefly, the linear Ac-CLYHFC-NH_2_ peptide was synthesized through solid-phase peptide synthesis using a Tribute automated synthesizer on Rink Amide MBHA resin (Gyros Protein Technologies, Tucson, AZ, USA). Cleavage from the solid support was performed using a TFA-based cocktail (90% TFA, 5% saturated phenol, 2.5% triisopropylsilane, 2.5% water) for 2 h with stirring. In solution, the crude peptide was precipitated in cold diethyl ether, followed by isolation with centrifugation for 5 min at 3500 rpm. The dried crude peptide was then dissolved in acetonitrile/water and was purified using a semipreparative HPLC system with a C-18 column (Waters xBridge; 250 mm × 19 mm; 5 μM particle size; Waters Corporation, Milford, MA, USA). The peak of interest was characterized using ESI-MS^+^ mass spectrometry and an analytical HPLC system (Agilent 1100 with microsorb-MV-100-5 C-18 column; 250 mm × 4.6 mm; 5 μm particle size, Agilent Technologies, St. Clara, CA, USA). Fractions containing the linear peptide were pooled, high-diluted in 1 L ammonium bicarbonate buffer pH = 8.5, and cyclized using air bubbling overnight to form the disulfide bond. The cyclic peptide Cyclo(1,6)-Ac-CLYHFC-NH_2_ was obtained as a white powder with 96% relative purity after solvent removal under vacuum and repurification using a semi-preparative HPLC system.

### 4.2. NMR Spectroscopy

The Cyclo(1,6)-Ac-CLYHFC-NH_2_ peptide (6 mg) was dissolved in 600 µL DMSO-d_6_. One-dimensional and two-dimensional (2D) NMR experiments were carried out on a 500 MHz Bruker AVIII spectrometer equipped with a cryogenically cooled carbon observe probe. Proton resonance assignments were determined using ^1^H-Correlated spectroscopy (COSY) and total correlation spectroscopy (TOCSY). Spectra processing including baseline, phase correction, visualization, and integration were performed using Mestrenova 14.2.0 software (Mestrelab Research S.L., Santiago, Spain). The interproton distances were determined using nuclear Overhauser effect spectroscopy (NOESY) spectra. Interproton distances (*d_ij_*) were calculated using NOE cross-peak intensity using the distance of Phe5 geminal methylene protons, which separated by 1.78 Å as the reference (*d_ref_*), according to the relation [20]:

(1) $$\frac{I_{ref}}{I_{ij}}=\frac{{d_{ij}}^{6}}{{d_{ref}}^{6}}$$

where *d_ij_* = the interproton distance; *d*_ref_ = 1.78 Å; *I_ref_* = the NOE intensity of geminal methylene protons of Phe5; and *I_ij_* = NOE intensity of the interactive protons.

The temperature-dependent amide proton chemical shift was observed by one-dimensional 1H experiment between 293 K and 323 K with a 5 K increment to define the NH proton temperature coefficient. The ^3^J_NH-HCα_ scalar coupling constant was calculated from the backbone amide region on the ^1^H spectra at 298 K, and the correlation between the coupling constant and the dihedral angle restraints was predicted using parameterized Karplus Equation (2), where θ = |φ − 60°| [25,29].

(2) JNH−HCa3=9.4 cos2θ−1.1 cosθ+0.4

### 4.3. Computational Simulation

Initial peptide backbone and side chain coordinates for the MD simulation of Cyclo(1,6)-Ac-CLYHFC-NH_2_ were obtained through de novo flexible docking of the cyclic peptide against MTB RpoB (PDB ID: 5UHC) using Autodock Crankpep (ADFRSuite 1.7) [31]. *N*-terminus acetyl capping was performed using YASARA View molecular graphic software for Windows, a part of openbabel 2.3.2 distribution [33]. The S-S bond was introduced through a brief minimization step in vacuum with GROMACS 5.1.4 to bring the S-S atoms distance to <0.25 nm while defining the terminus to none/CT2 to add the amidated end.

MD simulations were carried out using the GROMACS 5.1.4 program with CHARMM forcefield. The system was built in a 3.716 nm cubic box, solvated with a TIP3P water model, and neutralized to achieve 0.15 M ionic concentration. After system minimization, a 10 ns NPT equilibration was performed at 298 K and 1 atm. The full MD trajectories were generated for 100 ns, with a 2 fs timestep. For NMR-constrained MD simulations, simple distance restraints using conservative restraints force and default force constants were set for the parameter file. Backbone NOE restraints were included in the topology file as a range from the experimental distance, and the φ angles restraints were added for Leu2, Tyr3, and His4 residues. For the analysis of the solution structure, all superpositions and RMSD calculations were performed against the backbone atoms (N, Cα, C) for each residue. The starting coordinate for the simulation was used as the reference for the calculation. Clustering was performed using *gmx cluster* with a 0.05 Å cut-off. Visualization was performed using the molecular graphic program UCSF Chimerax 1.13.3 [36].

## 5. Conclusions

In summary, Cyclo(1,6)-Ac-CLYHFC-NH_2_ has a stable conformation in solution with a stable βI-turn at Cys1-Leu2-Tyr3-His4 as determined by NMR and MD simulations. The temperature-dependent NMR experiment showed the possibility of an intramolecular hydrogen bond network between the backbone and acetyl residue and identified a solvent-accessible amide proton for future modification. Using molecular docking and MD simulations, this conformation will be used to design selective derivatives that selectively and tightly bind to the RIF-binding site on MTB RNA polymerase. The derivatives will be evaluated in inhibiting MTB RNA polymerase activity.

## Figures and Tables

**Table 1:** Proton chemical shifts of Cyclo(1,6)Ac-CLYHFC-NH_2_ measured in DMSO-d_6_ at 298 K.

Residue	Chemical Shift (δ)			
	HN	Hα	Hβ	Other
Ac				1.85 (CH_3_)
Cys-1	8.23	4.62	2.80, 3.28	
Leu-2	8.24	4.06	1.31	1.40 (Hγ); 0.75, 0.80 (Hδ1, Hδ2)
Tyr-3	7.96	4.20	2.77, 3.01	6.97 (Ar. H2, H6); 6.64 (Ar. H3, H5); 9.19 (OH)
His-4	8.38	4.36	3.09	8.95 (Ar. H2), 6.98 (NH), 7.29 (Ar. H4)
Phe-5	8.28	4.40	2.92, 3.17	7.12 (Ar. H2, H6); 7.26 (Ar. H3, H5)
Cys-6	8.38	4.19	3.07, 3.22	

**Table 2:** Temperature coefficient calculation.

Residue	Δδ/ΔT (ppb/K)	Equation	R^2^
Cys1	4.43	y = 9.55 × 10^3^ − 4.43x	0.99
Leu2	2.57	y = 9.01 × 10^3^ − 2.57x	0.99
Tyr3	1.64	y = 8.45 × 10^3^ − 1.64x	0.97
His4	6.21	y = 1.02 × 10^4^ − 6.21x	1.00
Phe5	5.86	y = 1 × 10^4^ − 5.86x	0.99
Cys6	3.86	y = 9.53 × 10^3^ − 3.86x	0.99

**Table 3:** Interproton distance calculation.

Cross-Peaks	HN	HN	HCα	HCα	HCβ	HCβ	HCδ	Others	Intensity	Distance
NH-NH	Leu2	Tyr3							0.2872	2.12
	Tyr3	His4							0.1752	2.30
NH-HCα	Cys1		Cys1						0.7648	1.80
	Leu2		Leu2						0.2047	2.24
	Tyr3		Tyr3						0.2278	2.20
	His4		His4						0.8717	1.76
	Phe5		Phe5						0.2474	2.17
	Cys6		Cys6						0.9081	1.75
	Leu2		Tyr3						0.2318	2.20
	Leu2		His4						0.1126	2.48
	Phe5		Cys6						0.3367	2.06
	Cys1		His4						0.0443	2.89
	Cys1		Tyr3						0.0315	3.06
NH-HCβ	Cys1				Cys1				0.3367/0.2247	2.06/2.21
	Leu2				Leu2				0.2691	2.14
	Tyr3				Tyr3				0.1416/0.0778	2.38/2.63
	His4				His4				-	-
	Phe5				Phe5				0.1114	2.48
	Cys6				Cys6				-	-
	Leu2				Tyr3				0.0858	2.59
	His4				Phe5				0.1477	2.37
	Leu2				His4/Cys6				0.0232	3.22
	Tyr3				His4/Cys6				0.1905	2.27
HCα-HCα			Cys1	Leu2					0.0377	2.97
			Cys1	His4					0.2369	2.19
			Phe5	Cys6					0.0857	2.59
HCα-HCβ			Cys1		Cys1				0.1782	2.29
			Leu2		Leu2				0.2842	2.12
			Tyr3		Tyr3				0.1998	2.25
			His4		His4				0.2215	2.21
			Phe5		Phe5				0.1881	2.27
			Cys6		Cys6				0.6672	1.84
			Cys1		Leu2				0.0439	2.90
			Cys1		His4				0.1161	2.46
			Leu2		Tyr3				0.0302	3.08
HCβ-HCβ					Cys1	Cys1			1.1342	1.69
					Tyr3	Tyr3			0.9626	1.73
					Phe5	Phe5			0.8462	1.77
Others			Leu2				Leu2		0.1588/0.0482	2.34/2.85
			Cys1					Ac	0.0284	3.12
	Cys1							Ac	0.4131	1.99
	Leu2						Leu2		0.0434	2.90

**Table 4:** Coupling constant and dihedral angle calculation.

Residue	^3^J_NH-HCα_	X1	X2	θ1	θ2	φ1		φ2	
Cys1	5.74	0.8145	−0.6975	35.46	134.23	95.46	24.54	−74.23	−165.77
Leu2	5.12	0.7695	−0.6525	39.69	130.73	99.69	20.31	−70.73	−169.27
Tyr3	8.51	0.9892	−0.8722	8.43	150.71	68.43	51.57	−90.71	−149.29
His4	7.63	0.9375	−0.8205	20.36	145.13	80.36	39.64	−85.13	−154.87
Phe5		Incalculable							
Cys6	7.63	0.9375	−0.8205	20.36	145.13	80.36	39.64	−85.13	−154.87

**Table 5:** Dihedral angles measured on the top clusters from MD simulations.

Residue	NOE-Restrained		Unrestrained	
	φ	ψ	φ	ψ
Cys1	-	−9.664	-	124.62
Leu2	−76.865	−26.501	−63.388	−39.216
Tyr3	−97.081	−12.468	−84.140	165.283
His4	−84.471	32.981	−67.761	138.508
Phe5	−93.392	−1.589	84.087	25.35
Cys6	−84.471	-	−85.510	-

## Data Availability

The original contributions presented in the study are included in the article/Supplementary Material, further inquiries can be directed to the corresponding author.

## Supplementary Materials

The following supporting material can be downloaded from https://www.mdpi.com/article/10.3390/ph17111545/s1: Figure S1: ^1^H NMR Spectra of Cyclo(1,6)Ac-CLYHFC-NH_2_; Figure S2: (^1^H-^1^H)-COSY Spectra of Cyclo(1,6)Ac-CLYHFC-NH_2_; Figure S3: TOCSY Spectra of Cyclo(1,6)Ac-CLYHFC-NH_2_; Figure S4: NOESY Spectra of Cyclo(1,6)Ac-CLYHFC-NH_2_; Figure S5: ^13^C Spectra of Cyclo(1,6)Ac-CLYHFC-NH_2_; Figure S6: (^1^H,^13^C)-HSCQ Spectra of Cyclo(1,6)Ac-CLYHFC-NH_2_; Figure S7: (^1^H,^13^C)-HMBC Spectra of Cyclo(1,6)Ac-CLYHFC-NH_2_; and Figure S8: ^1^H NMR Spectra of Ac-CLYHFC-NH_2_.
