# Supplementary material for: Structure of a Cyclic Peptide as an Inhibitor of Mycobacterium tuberculosis Transcription: NMR and Molecular Dynamics Simulations
Source: Pharmaceuticals (Basel). 2024 Nov 18;17(11):1545. doi: 10.3390/ph17111545 (PMC11597662; doi:10.3390/ph17111545)
Supplement: Supplementary file 1 [file pharmaceuticals-17-01545-s001.zip › pharmaceuticals-2792791-supplementary.pdf]

Supplementary Materials

Structure of a Cyclic Peptide as an Inhibitor of *Mycobacterium tuberculosis* Transcription: NMR and Molecular Dynamics Simulations

Figure S1. 1H NMR Spectra of Cyclo(1,6)Ac-CLYHFC-NH_2

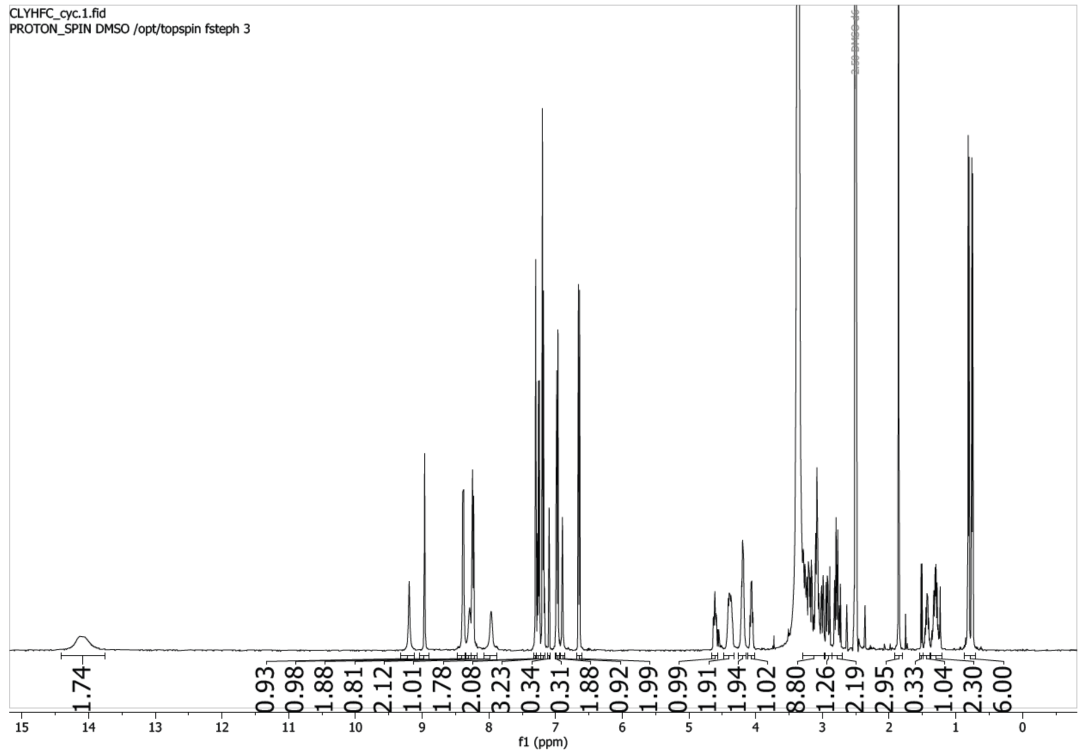

^{1}H NMR (500 MHz, DMSO) δ 14.11 (s, 2H), 9.20 (s, 1H), 8.96 (s, 1H), 8.39 (d, J = 7.7 Hz, 2H), 8.29 (s, 1H), 8.24 (dd, J = 8.1, 5.4 Hz, 2H), 7.96 (d, J = 8.1 Hz, 1H), 7.30 (s, 2H), 7.28 – 7.23 (m, 2H), 7.19 (d, J = 7.4 Hz, 3H), 6.99 – 6.94 (m, 2H), 6.90 (s, 1H), 6.69 – 6.61 (m, 2H), 4.61 (ddd, J = 12.0, 8.5, 3.9 Hz, 1H), 4.47 – 4.32 (m, 2H), 4.25 – 4.14 (m, 2H), 4.11 – 4.02 (m, 1H), 3.30 – 2.97 (m, 9H), 2.96 – 2.86 (m, 1H), 2.85 – 2.70 (m, 2H), 1.86 (s, 3H), 1.43 (dp, J = 13.2, 6.6 Hz, 1H), 1.38 – 1.21 (m, 2H), 0.78 (dd, J = 27.3, 6.5 Hz, 6H).

Figure S2. (^{1}H-^{1}H)-COSY Spectra of Cyclo(1,6)Ac-CLYHFC-NH_2

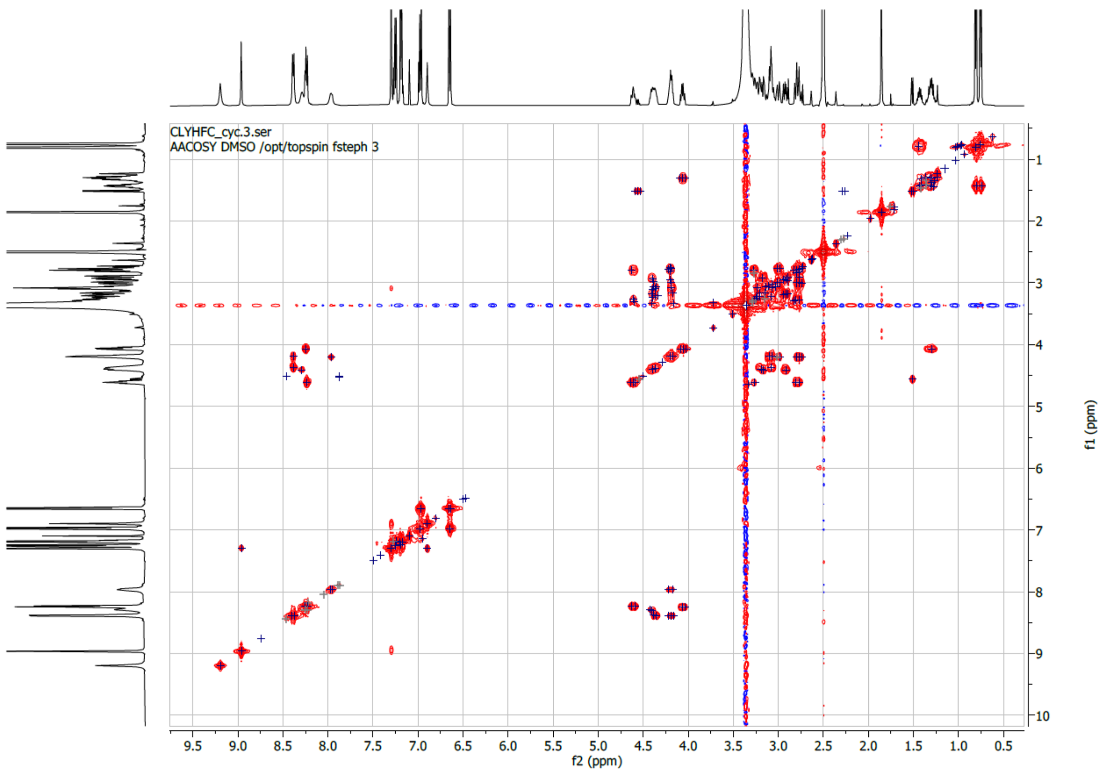

Figure S3. TOCSY Spectra of Cyclo(1,6)Ac-CLYHFC-NH_2

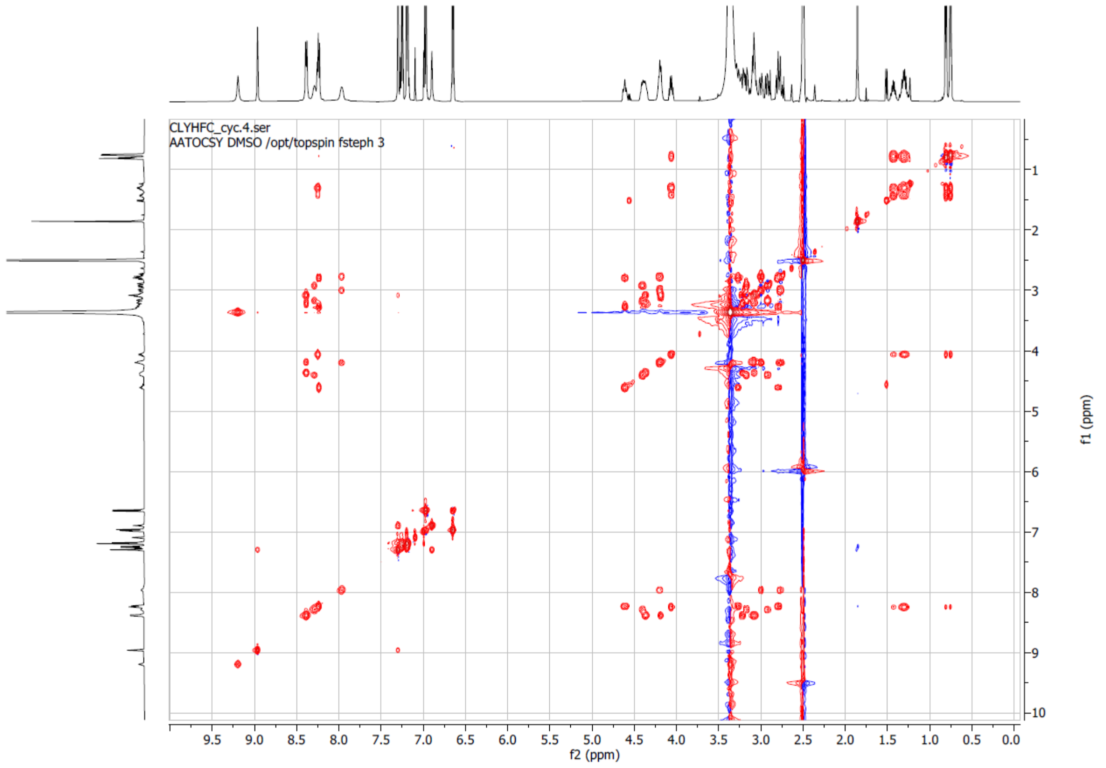

Figure S4. NOESY Spectra of Cyclo(1,6)Ac-CLYHFC-NH_2

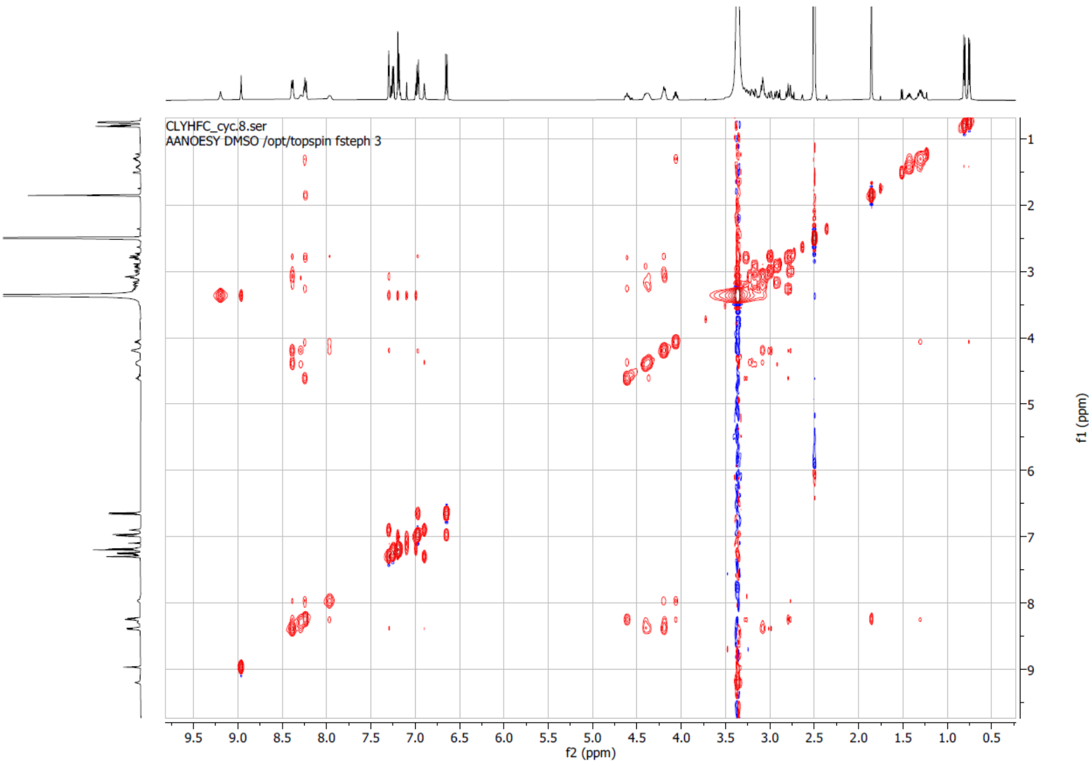

Figure S5. 13C Spectra of Cyclo(1,6)Ac-CLYHFC-NH_2

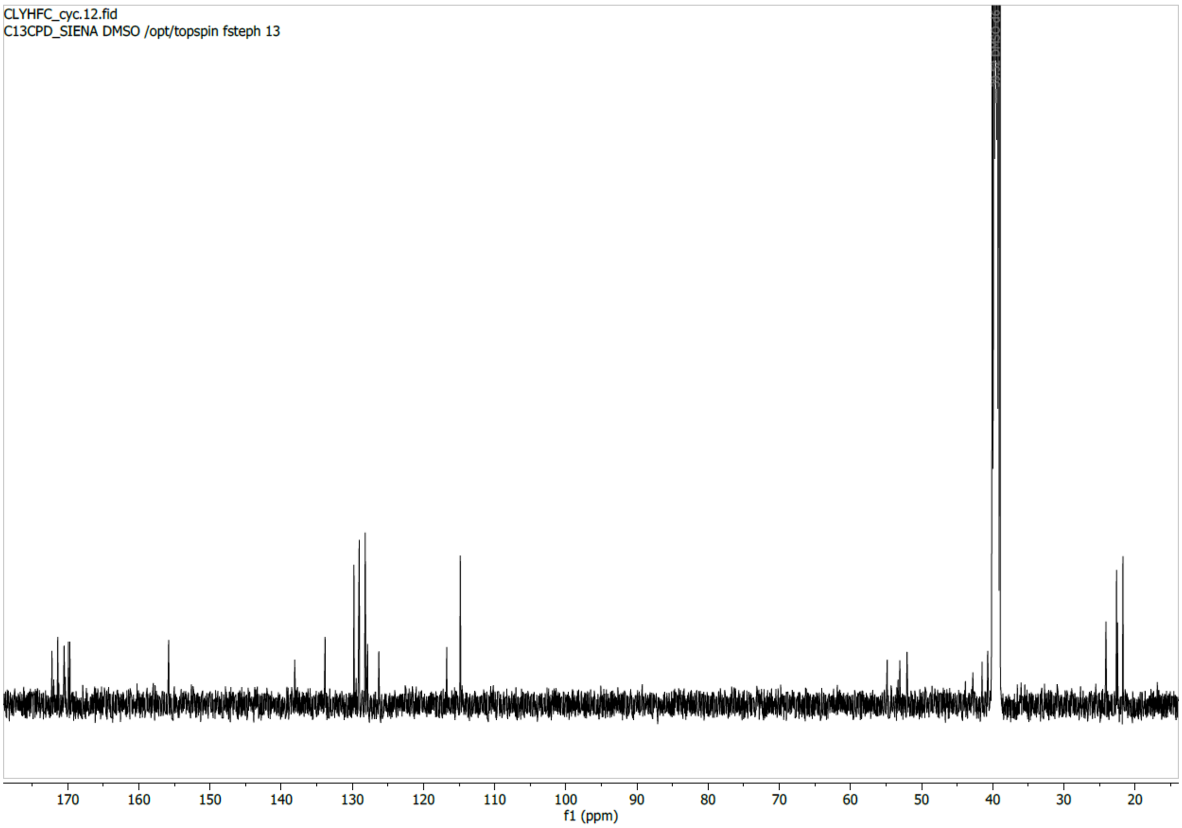

Figure S6. (^{1}H,^{13}C)-HSCQ Spectra of Cyclo(1,6)Ac-CLYHFC-NH$_2$

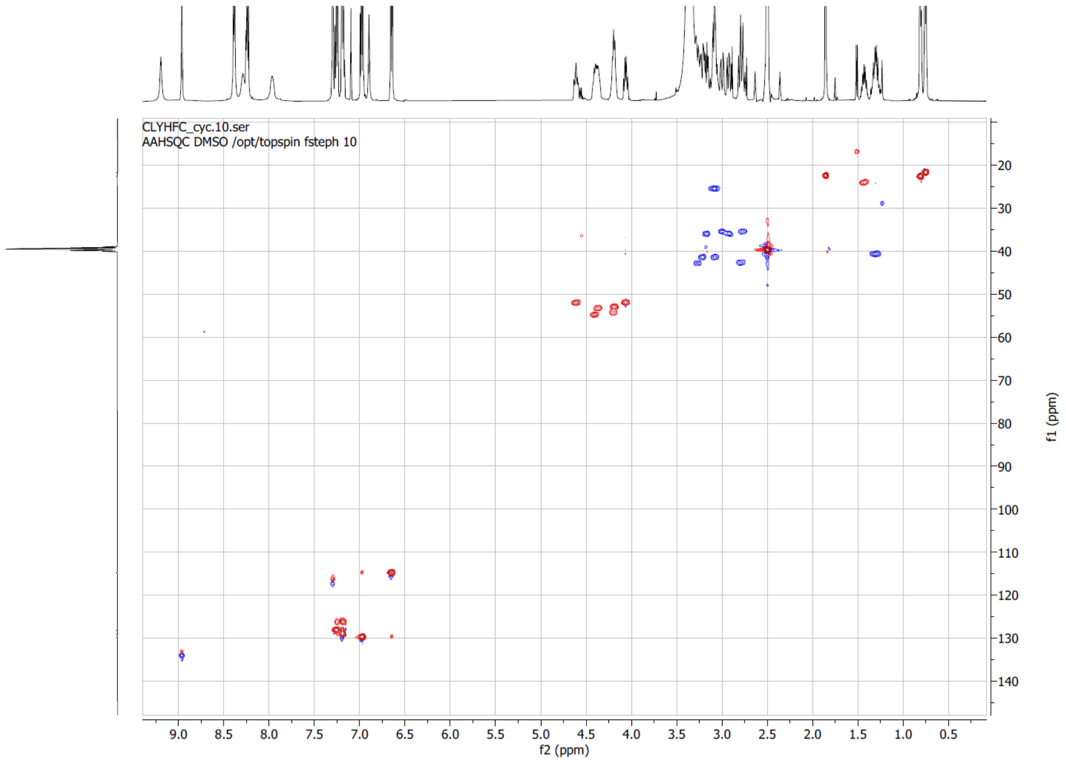

Figure S7. (^{1}H,^{13}C)-HMBC Spectra of Cyclo(1,6)Ac-CLYHFC-NH$_2$

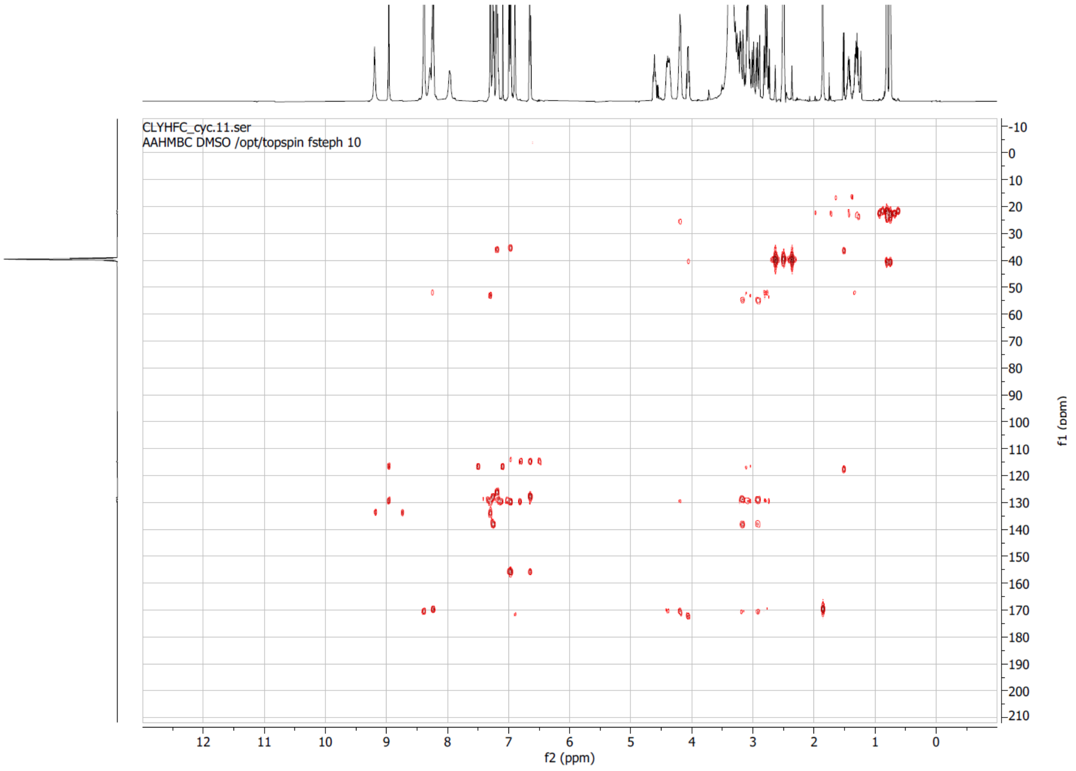

Figure S8. 1H NMR Spectra of Ac-CLYHFC-NH_2.

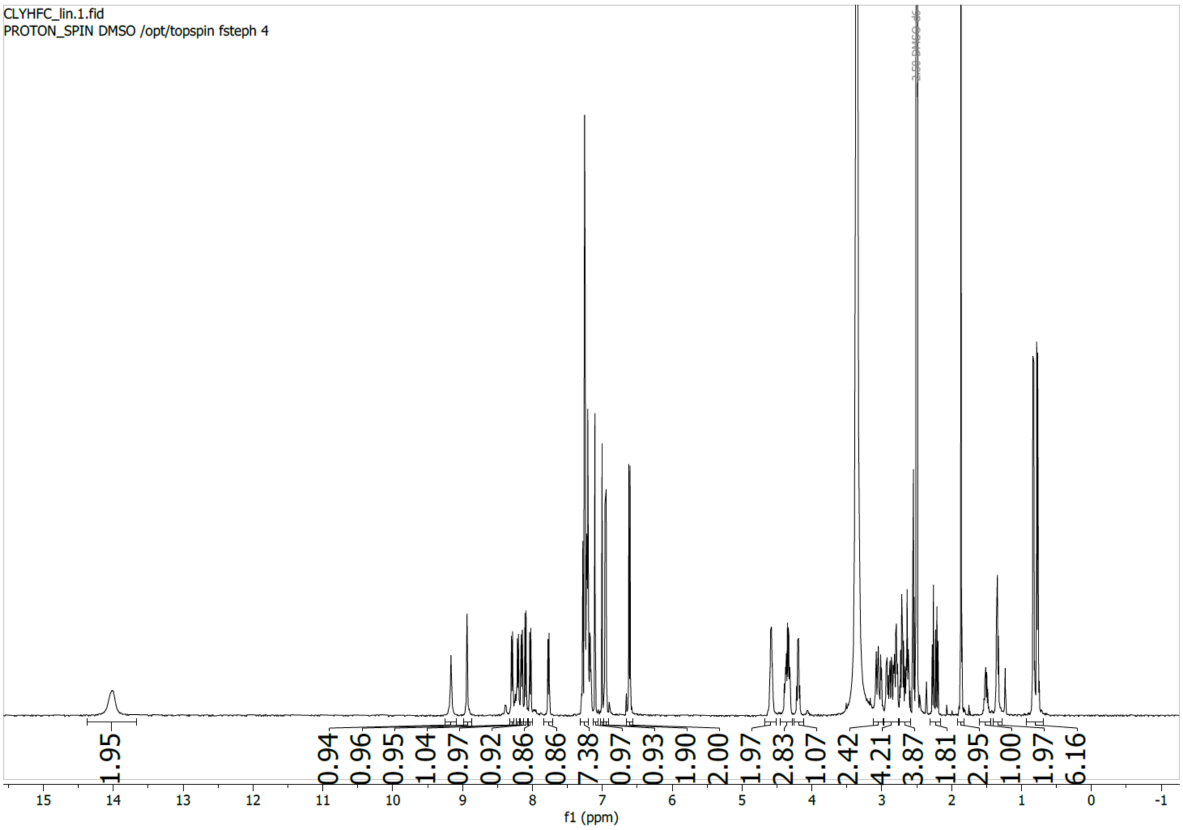

^{1}H NMR (500 MHz, DMSO) δ 14.01 (s, 2H), 9.17 (s, 1H), 8.94 (s, 1H), 8.29 (d, *J* = 8.1 Hz, 1H), 8.21 (d, *J* = 8.0 Hz, 1H), 8.15 (d, *J* = 7.5 Hz, 1H), 8.10 (d, *J* = 7.7 Hz, 1H), 8.03 (d, *J* = 8.0 Hz, 1H), 7.77 (d, *J* = 7.7 Hz, 1H), 7.32 – 7.20 (m, 7H), 7.11 (s, 1H), 7.00 (s, 1H), 6.98 – 6.91 (m, 2H), 6.65 – 6.56 (m, 2H), 4.67 – 4.52 (m, 2H), 4.45 – 4.28 (m, 3H), 4.20 (q, *J* = 7.7 Hz, 1H), 3.12 – 2.98 (m, 2H), 2.97 – 2.76 (m, 4H), 2.76 – 2.59 (m, 4H), 2.31 – 2.16 (m, 2H), 1.87 (s, 3H), 1.51 (dp, *J* = 13.4, 6.7 Hz, 1H), 1.35 (t, *J* = 7.3 Hz, 2H), 0.93 – 0.68 (m, 6H).
